# miR-17-5p promotes the invasion and migration of colorectal cancer by regulating HSPB2

**DOI:** 10.7150/jca.65614

**Published:** 2022-01-01

**Authors:** Weifang Yu, Jia Wang, Chao Li, Mingda Xuan, Shuangshuang Han, Yingfu Zhang, Pengfei Liu, Zengren Zhao

**Affiliations:** 1 Departments of Endoscopy Center, The First Hospital of Hebei Medical University, 89 Donggang Road, Shijiazhuang, 050031 Hebei, China; 2 Department of Internal medicine, The First Hospital of Hebei Medical University, 89 Donggang Road, Shijiazhuang, 050031 Hebei, China; 3 Hebei Medical University, 361 Zhongshan East Road, Shijiazhuang, 050017 Hebei, China; 4 Department of General Surgery, Hebei Key Laboratory of Colorectal Cancer Precision Diagnosis and Treatment, The First Hospital of Hebei Medical University, 89 Donggang Road, Shijiazhuang, 050031 Hebei, China

**Keywords:** Colorectal cancer, microRNA, miR-17-5p, HSPB2, RNA sequencing and data analysis

## Abstract

MicroRNA (miRNA) can affect tumor progression by regulating cell proliferation, apoptosis and metastasis. A significant upregulation of miR-17-5p expression was found in colorectal cancer (CRC) tissues by miRNA microarray chip analysis. However, the underlying mechanism of miR-17-5p in CRC is still unclear. The mRNA expression of miR-17-5p was significantly higher in CRC tissues than in adjacent normal tissues. In CRC group, the expression of miR-17-5p in cancer tissues with lymph node metastasis was higher compared with those without lymph node metastasis. The biological function of miR-17-5p was demonstrated through CCK-8, colony formation, flow cytometry and transwell assays. Overexpression of miR-17-5p inhibited CRC cell apoptosis, as well as promoting proliferation, migration and invasion. Transcriptome sequencing and miRNA target prediction software suggested that HSPB2 might be a target gene of miR-17-5p and luciferase reporter detection validated for the first time that miR-17-5p binds directly to the 3'-UTR of HSPB2. In the rescue experiment, the tumor suppressive effect of HSPB2 was detected and miR-17-5p could promote cell proliferation, migration and invasion by targeting HSPB2. Taken together, miR-17-5p promotes invasion and migration by inhibiting HSPB2 in CRC, thereby implicating its potential as a novel diagnostic biomarker and therapeutic target for CRC.

## Introduction

Colorectal cancer (CRC) is the third common malignancy worldwide associated with increased morbidity and mortality^1^. The five-year survival rate for patients with early CRC is 90%, as compared with 12% for advanced CRC^2^. In 2018, the International Agency for Research on Cancer (IARC) estimated that there would be more than 521,000 new cases and more than 248,000 new deaths of CRC in China, making it the major public health issue^3^. Although several oncogenes and tumor suppressor genes have been reported to be related to CRC, the molecular mechanism of CRC development remains poorly understood.

MicroRNAs (miRNA) are non-coding RNA species, generally 19-22 nucleotides in length, and can induce translation inhibition or cause mRNA degradation by specifically binding to the target gene^4^-^7^. It has been reported that miRNA regulates multiple vital cellular processes, including cell growth, differentiation, cycle progression, and apoptosis, and can be an essential biomarker for diagnosis and treatment of tumors^8^-^11^. miR-17-5p, a member of the miR-17-92 cluster, is located in the third intron on chromosome 13, which is an amplified genomic site in many cancers^12^, ^13^. Previously, miR-17-5p was found to be abnormally expressed in multiple cancers and is involved in the tumorigenesis and progression by inhibiting apoptosis and promoting proliferation and metastasis^14^-^21^. The previous analysis of miRNA microarray chips by our team has revealed a significant upregulation of miR-17-5p expression in CRC tissues than adjacent normal tissues^22^. However, the precise molecular mechanism through which miR-17-5p influences CRC progression remains largely unclear. Therefore, the effects that miR-17-5p could exert on tumorigenesis and CRC development, as well as the downstream target genes and interaction mechanisms of miR-17-5p are our prime concerns.

Heat shock protein B2 (HSPB2) is a member of the small heat shock protein (HSPs) family. It is expressed in the heart and skeletal muscle^23^. Most HSPB2 related studies have focused on Alzheimer's disease and muscular dystrophy^24^. In addition, the role of HSPB2 in tumors has also attracted the attention of researchers. The study has pointed out that HSPB2 is highly expressed in human breast cancer cells and is related to apoptosis resistance^25^. In addition, it has been found that HSPB2 is reduced or missing during the development of some tumors, such as pancreatic cancer and esophageal cancer^26^, ^27^. However, whether HSPB2 is also involved in regulating the development of colorectal cancer is not yet clear.

This study analyzed the expression level of miR-17-5p in CRC and explored its biological effects on CRC cells. It was confirmed for the first time that miR-17-5p directly targets and reduces the expression level of HSPB2 and plays an oncogenic miRNA role in the pathogenesis of colorectal cancer by regulating HSPB2. These findings indicate that the miR-17-5p-HSPB2 axis is an important regulator of the development of CRC and may provide a potential target for future CRC treatment.

## Materials and Methods

### Patients and specimens

Ninety-eight paired specimens of CRC tissues and adjacent normal tissues were provided by Clinical Biobank in the First Hospital of Hebei Medical University. Colorectal cancer and normal tissue were collected from surgical resection specimens of colorectal cancer patients. No patients had received radiotherapy or chemotherapy before surgery. All cancer tissues were confirmed histologically to be colorectal adenocarcinoma or mucinous carcinoma. They were immediately stored in liquid nitrogen within 5 minutes after resection and stored at -80°C for a long time. The research protocol was approved by the Ethics Committee of the First Hospital of Hebei Medical University, in line with the principles of the Declaration of Helsinki, and informed consent was obtained from each patient.

### Cell lines Culture

Human CRC cell lines HCT116, SW480, LOVO, SW1116, Caco2, and SW1463 cell lines were obtained from Prof. Jun Yu (The Chinese University of Hong Kong, Hong Kong, China). HCT116 were cultured in McCoy's 5A medium (Gibco, Gaithersburg, MD, USA) and the other cells lines were cultured in DMEM medium (Gibco, Gaithersburg, MD, USA, supplemented with 10% fetal bovine serum (FBS; Gibco, Gaithersburg, MD, USA) and 1% penicillin-streptomycin (Invitrogen, Carlsbad, CA, USA) in a 37°C humidified incubator with 95% air and 5% CO_2_.

### Transfection and plasmid construction

The hsa-miR-17-5p mimic, inhibitor, the respective negative control, HSPB2 overexpression plasmid or shRNA-HSPB2 (GeneCopoeia, MD, USA) were transfected into cells in 6-well plates using Lipofectamine 2000 (Invitrogen, Carlsbad, USA) when the cells were approximately 60-80% confluent. After the cells were cultured in medium without FBS for 6 h, the medium was replaced by that supplemented with 10% FBS. Cells were harvested 48 h after transfection and the effect of transfection was assessed by real-time quantitative polymerase chain reaction (qRT-PCR).

### RNA extraction and qRT-PCR

Total RNA was extracted from the transfected cells and tissues using Trizol Reagent (Invitrogen, Carlsbad, USA). To quantify miR-17-5p, All-in-One miRNA qRT-PCR kits (GeneCopoeia, MD, USA) were used with miR-17-5p-specific primers (HmiRQP0230, GeneCopoeia, MD, USA) according to the manufacturer's instructions. To quantify HSPB2, cDNA was reverse transcribed using PrimeScript RT reagent kit (Takara, Beijing, China). Then, a qRT-PCR was performed by using SYBR Green Master Mix (Vazyme, NJ, USA). U6 was used as internal control of miRNA, and GAPDH was used as internal control of mRNA. The primer sequences were as follows: HSPB2 (forward): 5′-ATGTCGGGCCGCTCAGTGCC-3′; HSPB2 (reverse): 5′-GTCACCTCGTCTGGGGTAAA-3′ 26; GAPDH (forward):5′-GAGTCAACGGATTTGGTCGT-3′; GAPDH (reverse) 5′-CATGGGTGGAATCATATTGGA-3′. All the experiments were performed in triplicate on an ABI7500 Sequence Detection System, and the mean cycle threshold (CT) data were obtained. The relative amount normalized to the internal control was calculated with the equation 2^-ΔΔCT^^28^.

### Cell apoptosis assay

The transfected cells were stained using the Annexin V-FITC/ PI Apoptosis Detection Kit (Beyotime, Jiangsu, China) in accordance with the manufacturer's instructions. 1 × 10^5^ cells were analyzed for apoptosis using a flow cytometry (BD, MA, USA), and the percentage of the apoptotic cells was quantified using Cell Quest software.

### Cell proliferation assay

The transfected cells were plated at 2 × 10^3^ cells per well in 96-well plates and incubated overnight in medium supplemented with 10% FBS. Cell proliferation was measured using counting kit-8 (CCK-8) reagent (Dojindo, Tokyo, Japan) at 24, 48, and 72 h post-transfection following the manufacturer's instruction. The absorbance at 450 nm was measured using a Promega GloMax Luminescence detector (Promega, MD, USA).

### Colony formation assay

1 × 10^3^ transfected cells were plated in cultured dishes and grown for 2 weeks. Subsequently, the cells were washed twice with phosphate buffer saline (PBS), fixed with 4% paraformaldehyde and stained with 0.5% crystal violet for 1 h. The dishes were scanned and the average number of colonies was achieved.

### Transwell invasion and migration assay

Invasion and migration assay were performed using Transwell chambers with 8-μm pores (Corning, MA, USA). For invasion assay, 2 × 10^5^ transfected cells suspended in 500 μl serum-free medium were placed in the upper chambers coated with Matrigel, and medium containing 10% FBS was added to the lower chamber as a chemoattractant. For migration assay, cells in 100 μl serum-free medium were added to the upper chambers, and medium containing 10% FBS was placed in the lower chamber as a chemoattractant. Following incubation at 37°C for 24 h, cells on the upper surface of the chamber were gently wiped off with cotton swabs. The cells were stained using a Diff-Quick stain kit according to the manufacturer's protocol and counted blindly (three random fields per chamber).

### RNA sequencing and data analysis

Total RNA was extracted from cells transfected with hsa-miR-17-5p mimic or inhibitor and the negative control cells, and then subjected to commercial RNA-sequencing (RNA-seq) analysis (Novogene). Preparation of library and sequencing of transcriptome were performed using NEB Next Ultra RNA Library Prep Kit for Illumina (Novogene, Beijing, China) following the manufacturer's protocols, and the attribute sequence of each sample was added with an index code. The clustering of the index-coded samples was achieved on a cBot Cluster Generation System. After cluster generation, 125-bp paired-end reads to genes were mapped using HTSeq v0.6.0 software. Afterwards, fragments per kilobase of transcript per million fragments mapped (FPKM) were also calculated. Analysis of differential expression was performed using the DEGSeq R package through the one scaling normalized factor. Corrected P values of 0.005 and log2 (fold changes) of 1 were set as the threshold for significantly differential expression after adjusted using the Benjamini-Hochberg method. Moreover, the hierarchical clustering and heatmaps were generated to show the normalized expression among the samples.

### Luciferase reporter assay

The 3'-UTR sequence of HSPB2 predicted to interact with miR-17-5p within the target sites was synthesized and inserted into psiCHECK2 vector (Sangon Biotech, Shanghai, China). Among them, four sets of vectors contain only one potential binding site, and one set contains all four sets of binding sites. For the luciferase reporter assays, HCT116 and LOVO cells were cultured in 96-well plates, and each well was co-transfected with firefly luciferase reporter plasmids, miR-17-5p mimic, inhibitor or the respective negative control using Lipofectamine 2000 (Invitrogen, Carlsbad, USA). The firefly luciferase and renilla luciferase activities were evaluated using the Dual Luciferase Reporter Assay Kit (Promega, MD, USA) following the manufacturer's instructions after transfection for 48 h.

### Western blot analysis

Total protein was isolated from the cultured cells using RIPA lysis buffer (Beyotime, Shanghai, China). Proteins were separated by 12% SDS-PAGE (Bio-Rad) and transferred onto polyvinylidene difluoride membranes (Millipore, MA, USA). The immunoreactive protein bands were detected by the Odyssey Scanning System (Li-Cor, Lincoln, USA) after using antibodies against HSPB2 (Proteintech, Wuhan, China) and β-actin (Solarbio, Beijing, China).

### Databases analysis

We used the online databases TargetScan^29^, microRNA.org^30^ and mirtarbase^31^ to predict target genes of miR-17-5p, and confirmed the specific binding sites for miR-17-5p on the 3'-UTR region of the target gene. In addition, the online database The Cancer Genome Atlas (TCGA) and Gene Expression Profiling Interactive Analysis (GEPIA) were used to further ascertain the mRNA expression of the target gene.

### Statistical analysis

The results of normal distribution data are expressed as mean ± SD and the results of non-normal distribution data are expressed as median and quartile spacing. Student's t-test, one-way ANOVA and two-way ANOVA were used in this study. The cellular experiments were repeated three times. All statistical analyses were performed using GraphPad Prism 7 (GraphPad Software, CA, USA) and SPSS 21 (IBM, NY, USA). P<0.05 was considered statistically significant.

## Results

### miR-17-5p was upregulated in most of the CRC tissues and cells

**Figure 1:**
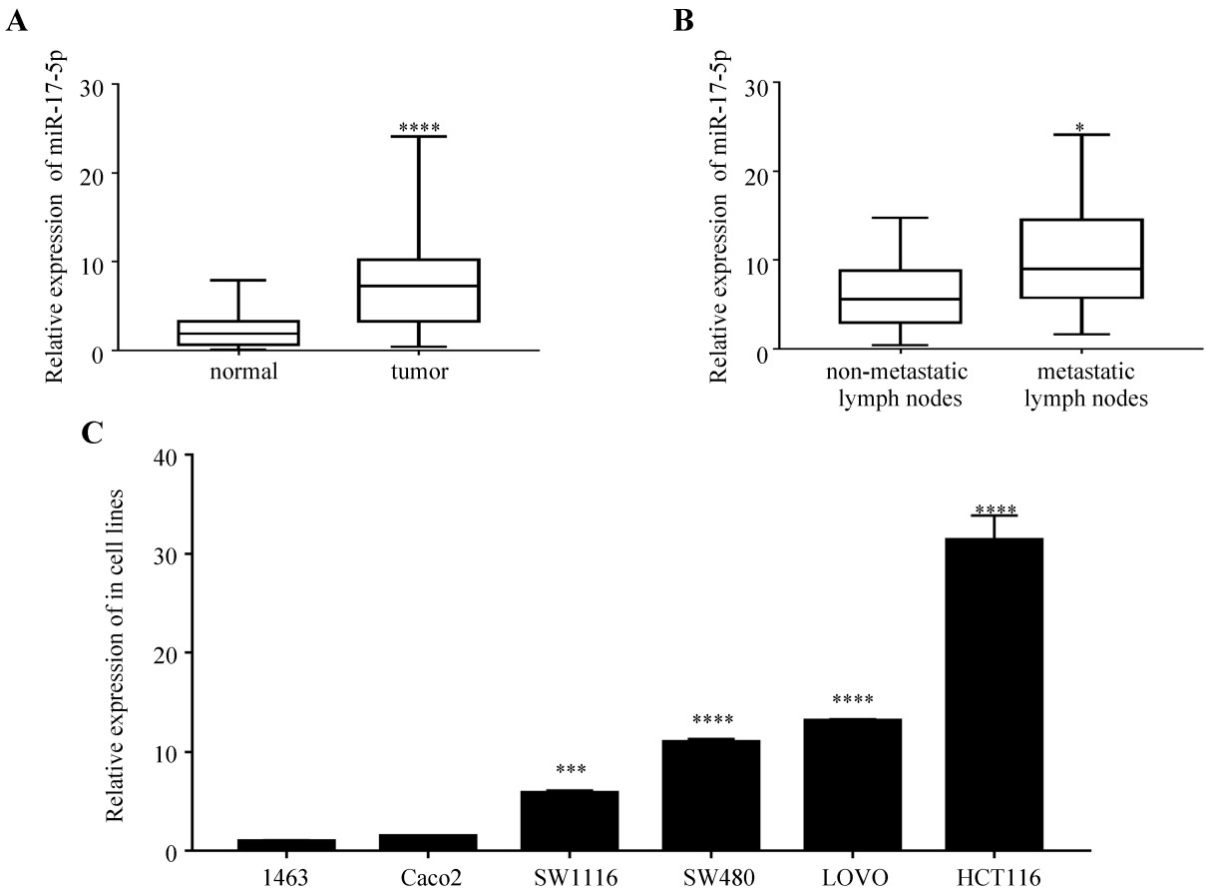
The expression of miR-17-5p is elevated in CRC patient samples and cell lines. (A) The expression level of miR-17-5p in 47 paired of CRC and adjacent normal tissues by qRT-PCR. (B) qRT-PCR analysis of miR-17-5p expression in CRC tissues with (n=18) and without lymph node metastasis (n=29). (C) Expression levels of miR-17-5p in 6 CRC cell lines. * P <0.05; *** P <0.001; **** P <0.0001.

We first examined the expression of miR-17-5p in 47 paired samples of CRC and the adjacent normal tissues. We found that miR-17-5p levels was significantly upregulated in CRC tissues compared to adjacent normal tissues (Figure 1A), which was consistent with our previous results by microarray chips in 4 pairs of CRC tissues. In the CRC group, the expression of miR-17-5p in cancer tissues with lymph node metastasis was higher compared to those without lymph node metastasis (Figure 1B), but it was not associated with clinicopathological features such as degree of differentiation, patient age, sex, tumor location and invasion (Table 1). In addition, high expression of miR-17-5p was found in most CRC cell lines (Figure 1C).

### Overexpression of miR-17-5p inhibited CRC cell apoptosis, and promoted proliferation, invasion and migration

**Figure 2:**
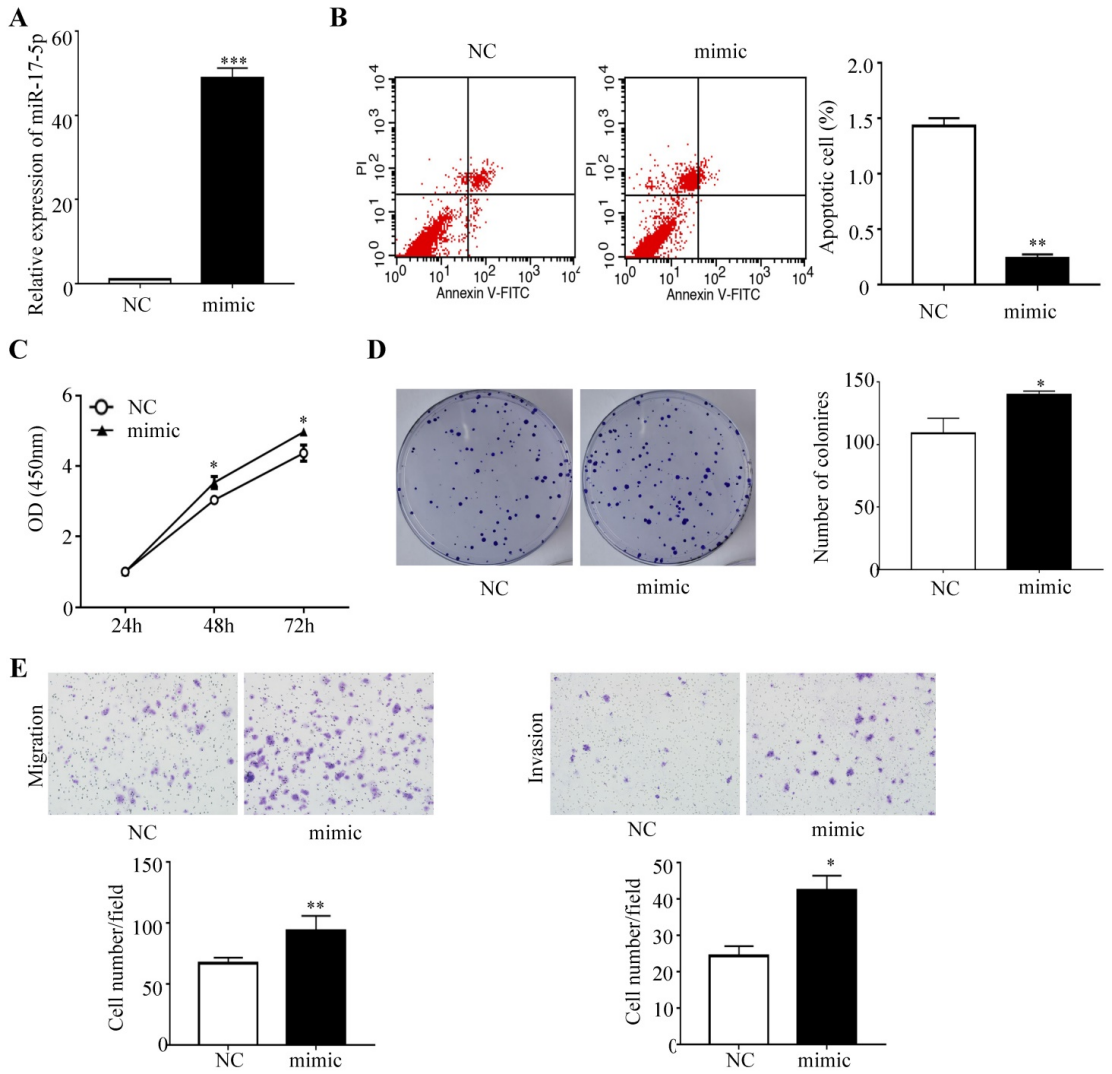
Overexpressed miR-17-5p inhibited CRC cell apoptosis, promoted the proliferation, invasion and migration. (A) The overexpression miR-17-5p was detected in HCT116 cells by qRT-PCR. (B) Flow cytometry was used to evaluate the cell apoptosis after increasing miR-17-5p expression. (C, D) CCK8 assay and colony formation assay were used to determine the effect of miR-17-5p overexpression on cell proliferation. (E) Transwell assay was performed to assess the migration and invasion potency after the expression of miR-17-5p was up-regulated. * P <0.05; ** P <0.01; *** P <0.001.

To determine whether miR-17-5p overexpression modulated CRC tumorigenesis, HCT116 cells with a certain miR-17-5p expression were transfected with miR-17-5p mimic and the negative control, and the overexpression effect was confirmed by qRT-PCR (Figure 2A). A flow cytometry assay showed that HCT116 cells overexpressing miR-17-5p had a significant reduction of apoptotic rate compared to control group (Figure 2B), as evident from the CCK8 assay that miR-17-5p overexpression induced the viability of CRC cells (Figure 2C). And colony formation assay revealed that the number of colonies was increased in cells overexpressing miR-17-5p (Figure 2D). Moreover, Transwell assay showed that over-expression of miR-17-5p resulted in a significant increase in the number of migrating and invading cells compared with control cells (Figure 2E).

### Silencing of miR-17-5p inhibited CRC cell proliferation, invasion and migration

**Figure 3:**
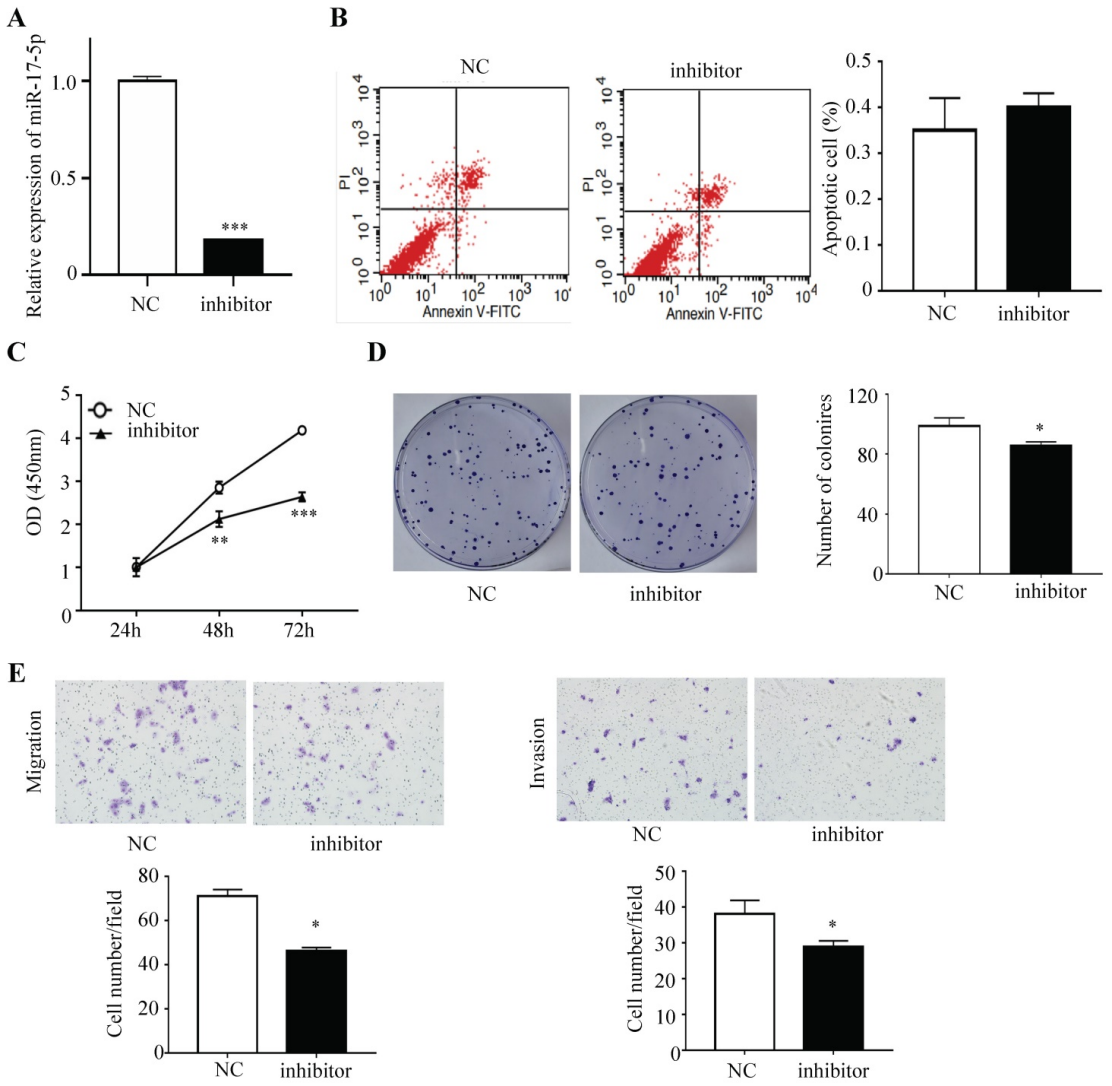
Endogenously reduced miR-17-5p inhibited CRC cell proliferation, invasion and migration. (A) The decrease expression of miR-17-5p was confirmed in HCT116 cells by qRT-PCR. (B) Flow cytometry was used to evaluate the cell apoptosis after reducing miR-17-5p expression. (C, D) CCK8 assay and colony formation assay were used to determine the effect of silence of endogenous miR-17-5p on cell proliferation. (E) Transwell assay was performed to assess the migration and invasion potency after the expression of miR-17-5p was down-regulated. * P <0.05; ** P <0.01; *** P <0.001.

HCT116 cells were subsequently transfected with inhibitor that could endogenously suppress the expression of miR-17-5p and the negative control, and a decreased expression of miR-17-5p was confirmed via qRT-PCR (Figure 3A). Apoptotic rate was not significantly decreased after the expression of miR-17-5p was suppressed, as compared with the control cells (Figure 3B). The CCK8 assay indicated that silencing endogenous miR-17-5p reduced the viability of CRC cells (Figure 3C). And colony formation assay showed that the number of colonies in miR-17-5p silenced cells was significantly reduced (Figure 3D). In addition, the silencing of endogenous miR-17-5p reduced cell migration and invasion compared to the control cells in the Transwell assay (Figure 3E).

### HSPB2 was a direct target of miR-17-5p

**Figure 4:**
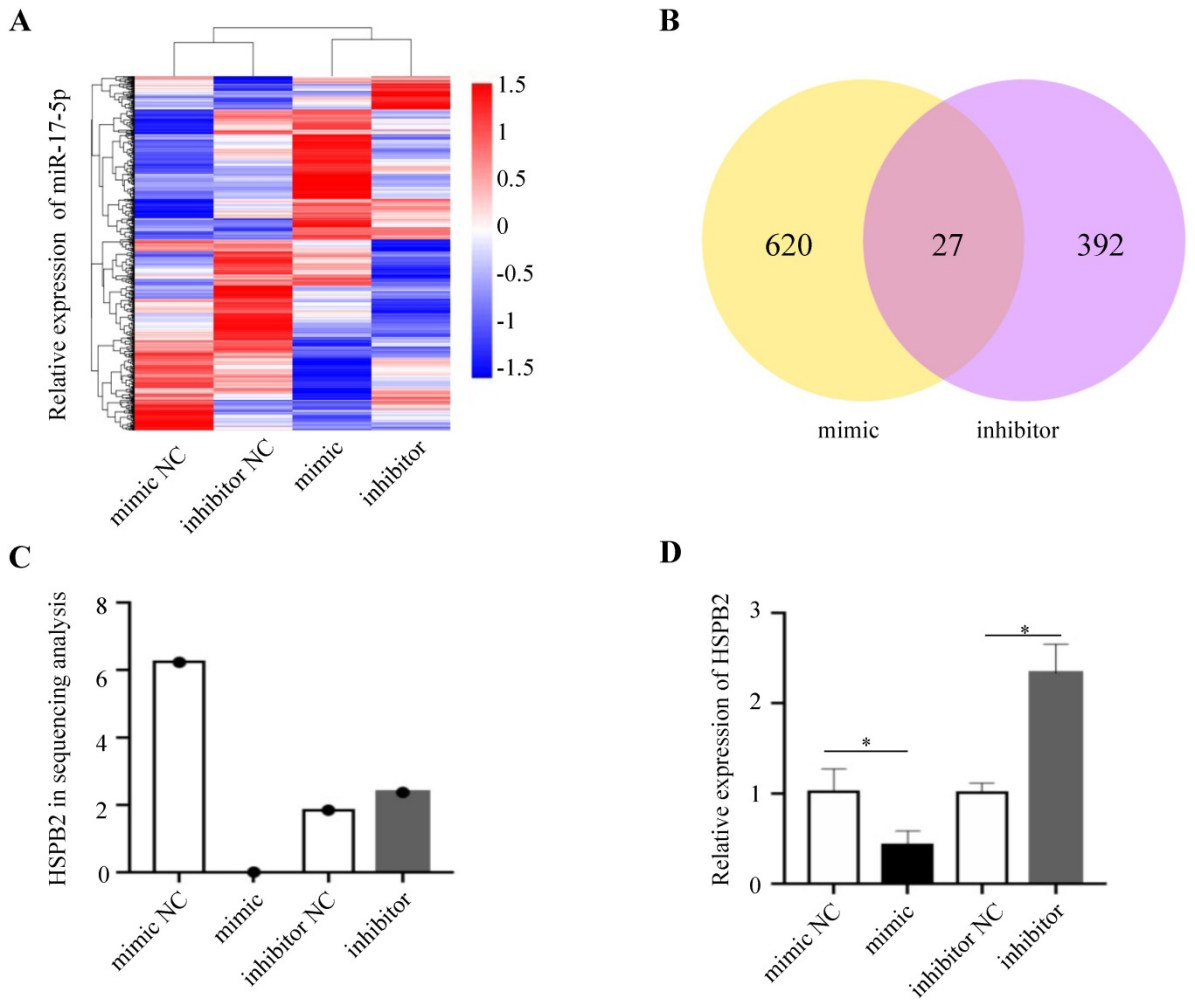
HSPB2 is predicted to be the target of miR-17-5p. (A) Hierarchical clustering heat map of RNA sequencing show all differential genes in up and down regulated miR-17-5p cells. (B) Venn diagram depicting the overlap differential genes in affected by miR-17-5p overexpression and knockdown. (C) Analysis data of HSPB2 expression in sequencing samples. (D) The relative expression of HSPB2 mRNA in sequencing samples by qRT-PCR. * P < 0.05

**Figure 5:**
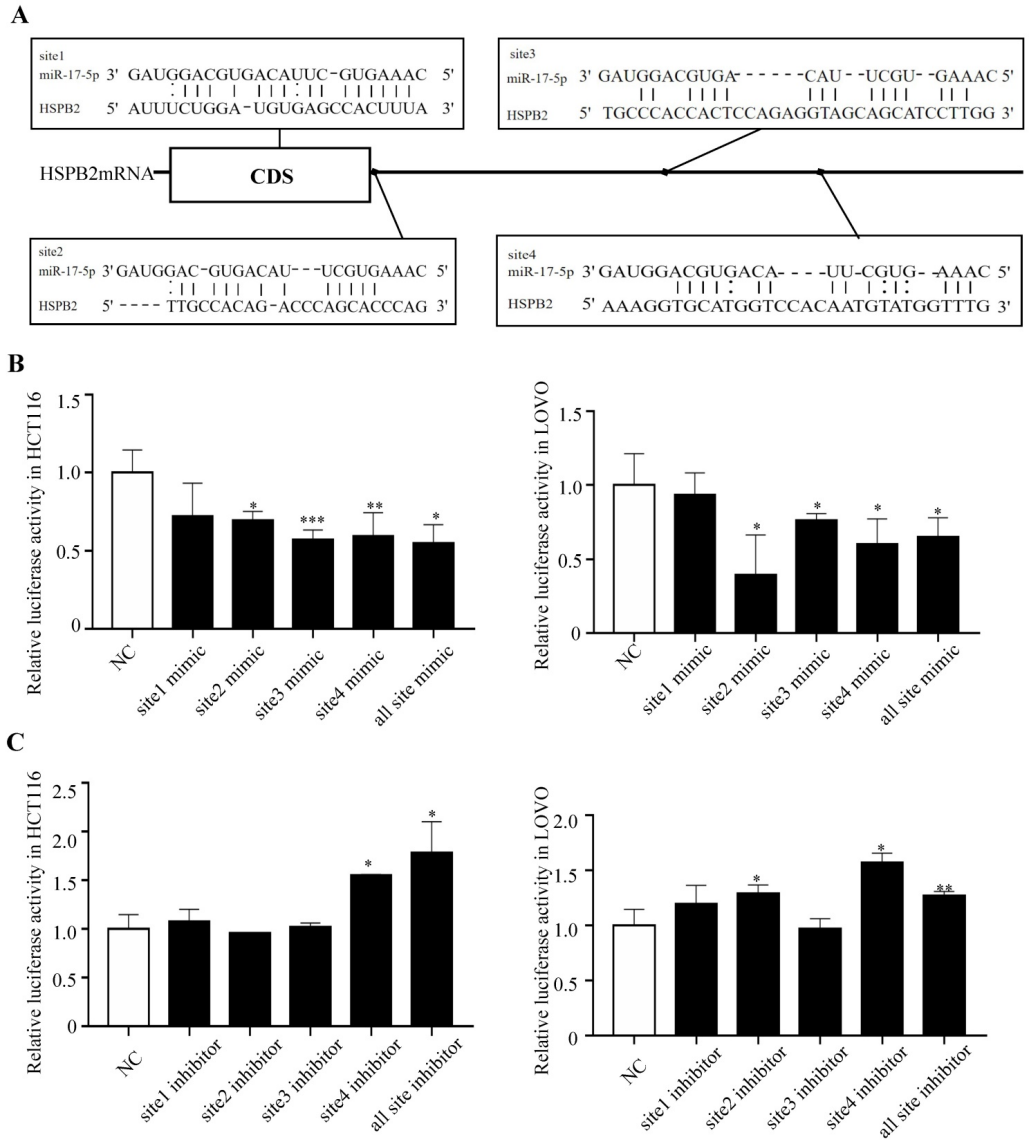
HSPB2 is identified as a direct target of miR-17-5p. (A) Predicted binding sites of miR-17-5p at the 3'-UTR of HSPB2 mRNA. (B) Relative luciferase activity in HCT116 and LOVO cells co-transfected with miR-17-5p mimics together with reporter vectors carrying HSPB2 3'-UTR binding sites. (C) Relative luciferase activity in HCT116 and LOVO cells co-transfected with miR-17-5p inhibitor together with reporter vectors carrying HSPB2 3'-UTR binding sites. * P <0.05; ** P <0.01; *** P <0.001.

In order to gain insight into the mechanism by which miR-17-5p acts as an oncogenic miRNA in CRC cells, we performed a transcriptome sequencing analysis to profile differentially expressed genes in HCT116 infected with miR-17-5p mimic and inhibitor. Significant difference revealed by RNA-seq analysis indicated global alterations in gene expression (Figure 4A). The mRNA expression of 647 and 419 genes was altered by overexpression or knockdown of miR-17-5p, respectively, of which 27 genes were regulated by both (Figure 4B). Many upregulated or downregulated genes might also be indirect targets, as they were affected as part of the regulatory network. Then we used three bioinformatics algorithms (TargetScan, microRNA.org, and mirtarbase) to screen whether there were potential miR-17-5p binding sites in these 27 genes. We found HSPB2 and miR-17-5p had opposite expression pattern changes according to the transcriptome sequencing analysis and qRT-PCR (Figure 4C-D). In addition, there are potential binding sites for miR-17-5p in the 3'-UTR region of HSPB2 to regulate translation or expression (Figure 5A). Therefore, these findings suggested that HSPB2 was a strong candidate for a direct and functional target gene of miR-17-5p in CRC. Then a luciferase reporter gene assay was performed using the constructed vector containing only one or all four sets of miR-17-5p potential binding sites. Compared to control group, the overexpression of miR-17-5p resulted in a significant decrease in luciferase reporter activity in the three sites and full-site group (Figure 5B), whereas miR-17-5p inhibitor led to a significant increase in reporter activity (Figure 5C).

### The HSPB2 mRNA was downregulated in CRC

**Figure 6:**
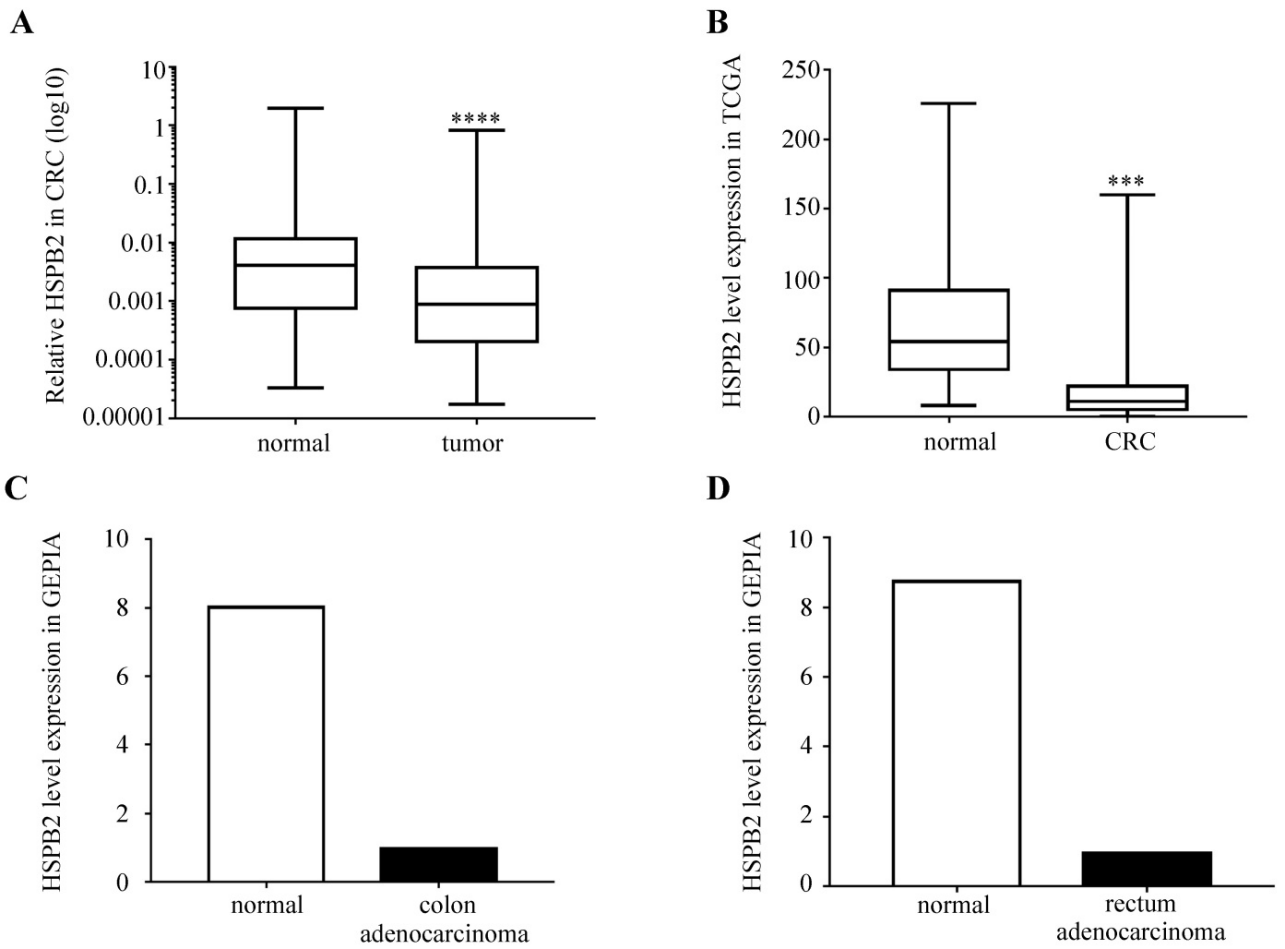
The expression of HSPB2 mRNA is down-regulated in CRC tissues. (A) The expression level of HSPB2 in 51 paired of CRC and adjacent normal tissues by qRT-PCR. (B) The HSPB2 expression profile of CRC tissues (n=647) and normal tissues (n=51) in CRC dataset of TCGA. (C) The HSPB2 expression profile of colon adenocarcinoma tissues (n=275) and normal tissues (n=349) in COAD dataset of GEPIA. (D) The HSPB2 expression profile of rectum adenocarcinoma tissues (n=92) and normal tissues (n=318) in READ dataset of GEPIA. *** P <0.001; **** P <0.0001.

We analyzed the expression levels of HSPB2 mRNA in 51 paired CRC and adjacent normal tissues. The results showed that the expression of HSPB2 in CRC tissues was significantly reduced compared to normal adjacent tissues (Figure 6A). Moreover, compared with normal tissues, the expression of HSPB2 mRNA in cancer tissues was significantly reduced according to the CRC dataset of TCGA, the COAD and READ datasets of GEPIA (Figure 6B-D).

### Interaction mechanism of miR-17-5p and HSPB2 in CRC

**Figure 7:**
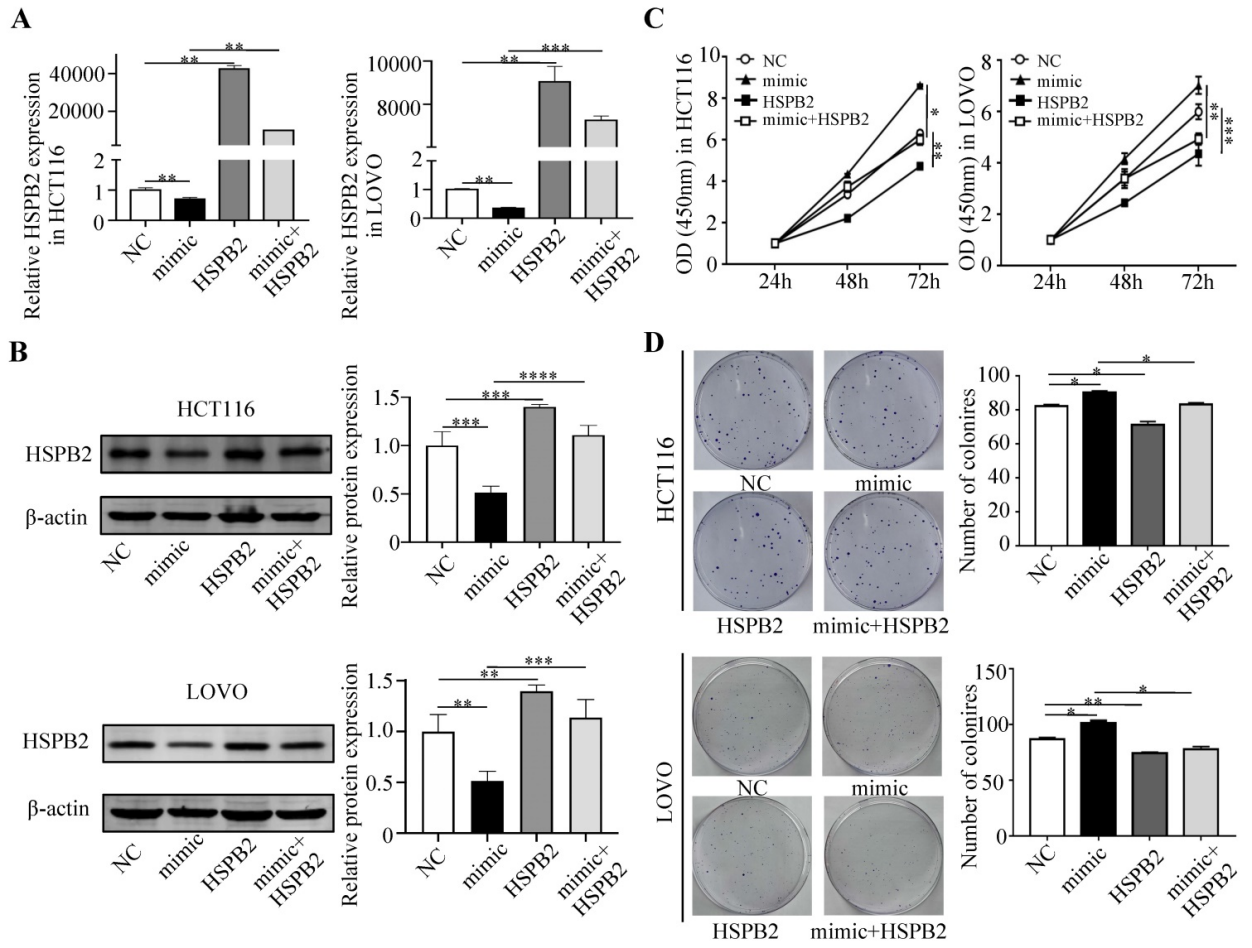
Reintroduction of HSPB2 could reverse the promotion of miR-17-5p in cell proliferation. (A, B) The overexpression of miR-17-5p and HSPB2 alone or simultaneously in HCT116 and LOVO cells confirmed by qRT-PCR and western blot analysis. (C, D) CCK8 analysis and colony formation assay were used to determine the effect of miR-17-5p and HSPB2 overexpression alone or simultaneously on cell proliferation. * P <0.05; ** P <0.01; *** P <0.001; **** P <0.0001.

**Figure 8:**
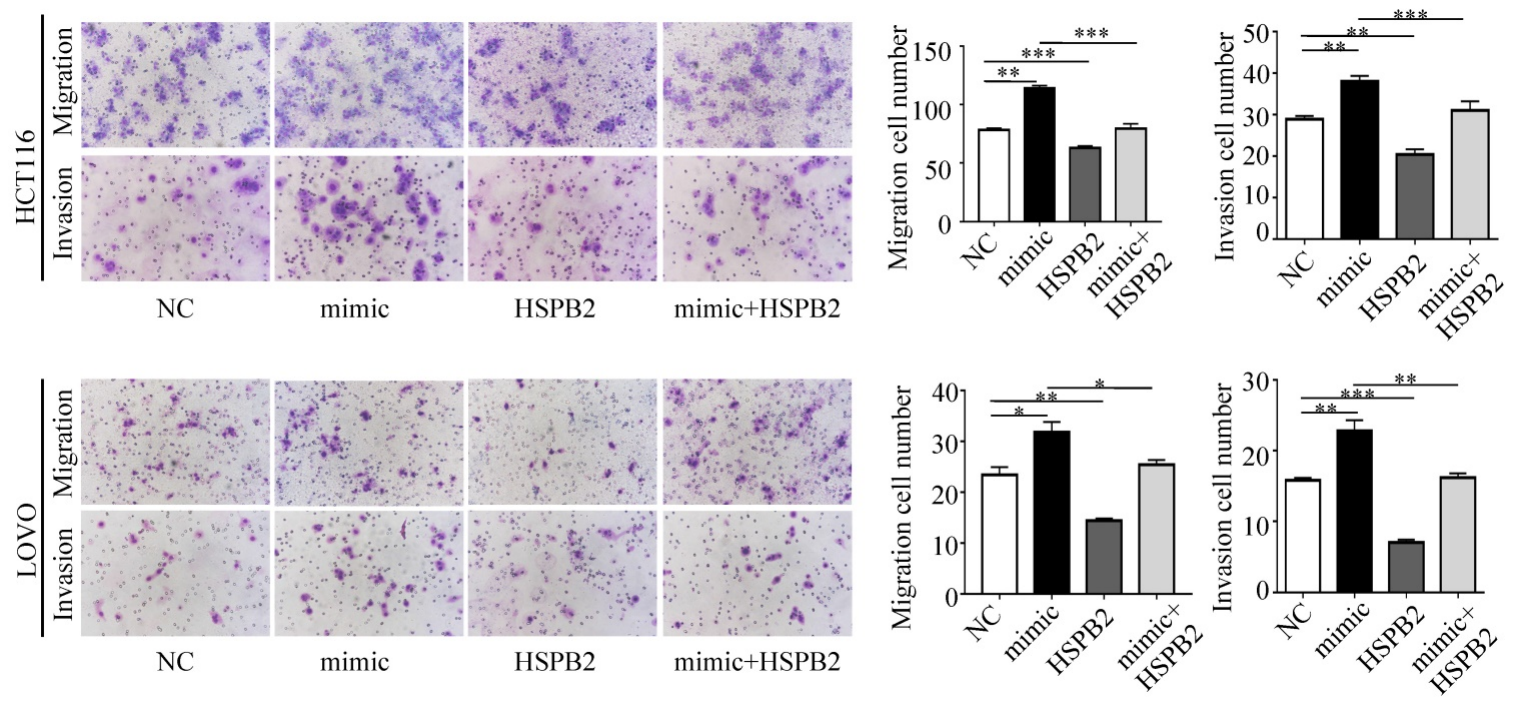
Transwell assay was performed to assess the migration and invasion potency after the expression of miR-17-5p and HSPB2 was up-regulated alone or simultaneously. * P <0.05; ** P <0.01; *** P <0.001.

**Figure 9:**
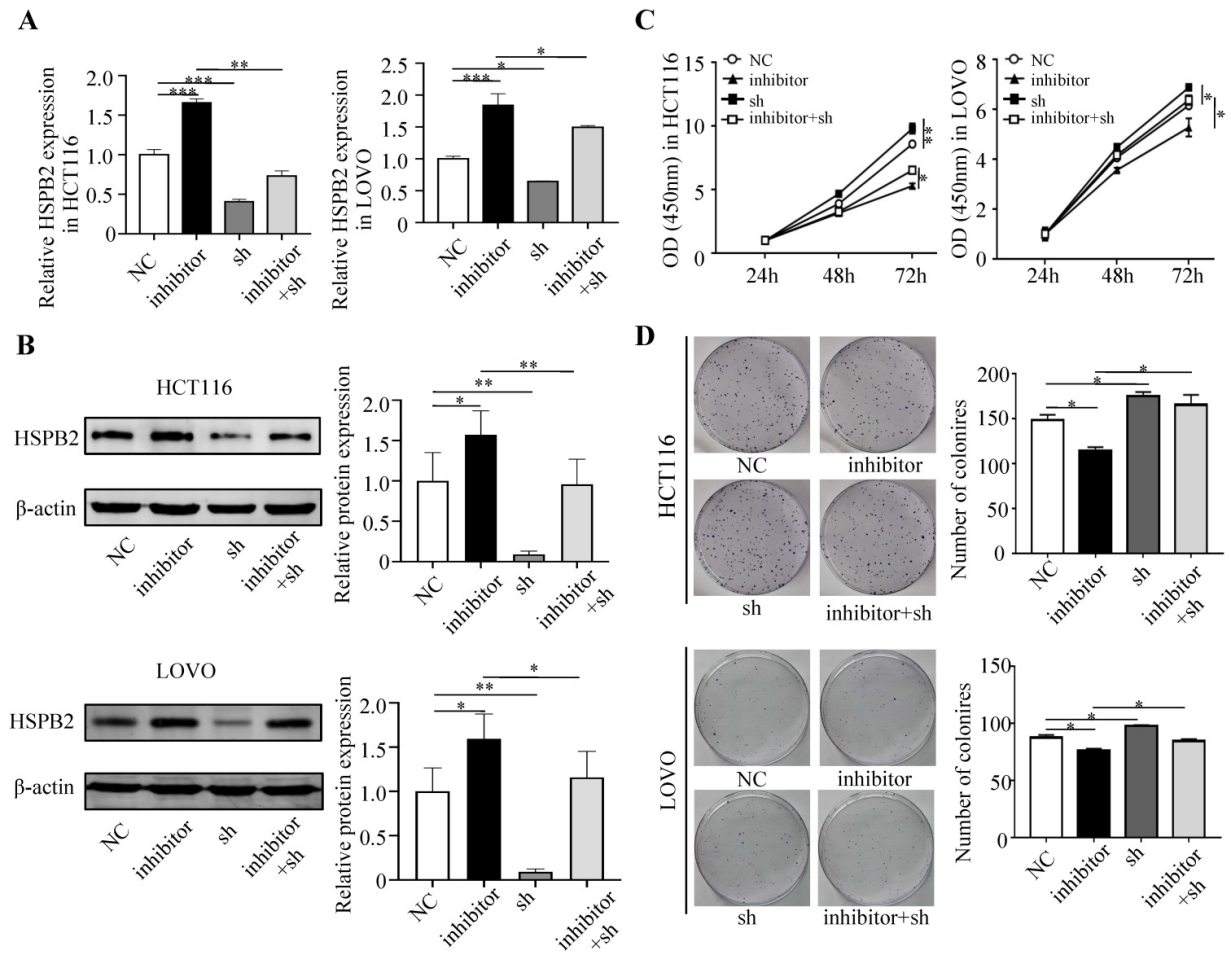
Reduced HSPB2 could rescue the suppression of miR-17-5p inhibitor in cell proliferation. (A, B) The decrease expression of miR-17-5p and HSPB2 alone or simultaneously in HCT116 and LOVO cells confirmed by qRT-PCR and western blot analysis. (C, D) CCK8 analysis and colony formation assay were used to determine the effect of knockdown miR-17-5p and HSPB2 alone or simultaneously on cell proliferation. * P <0.05; ** P <0.01; *** P <0.001.

**Figure 10:**
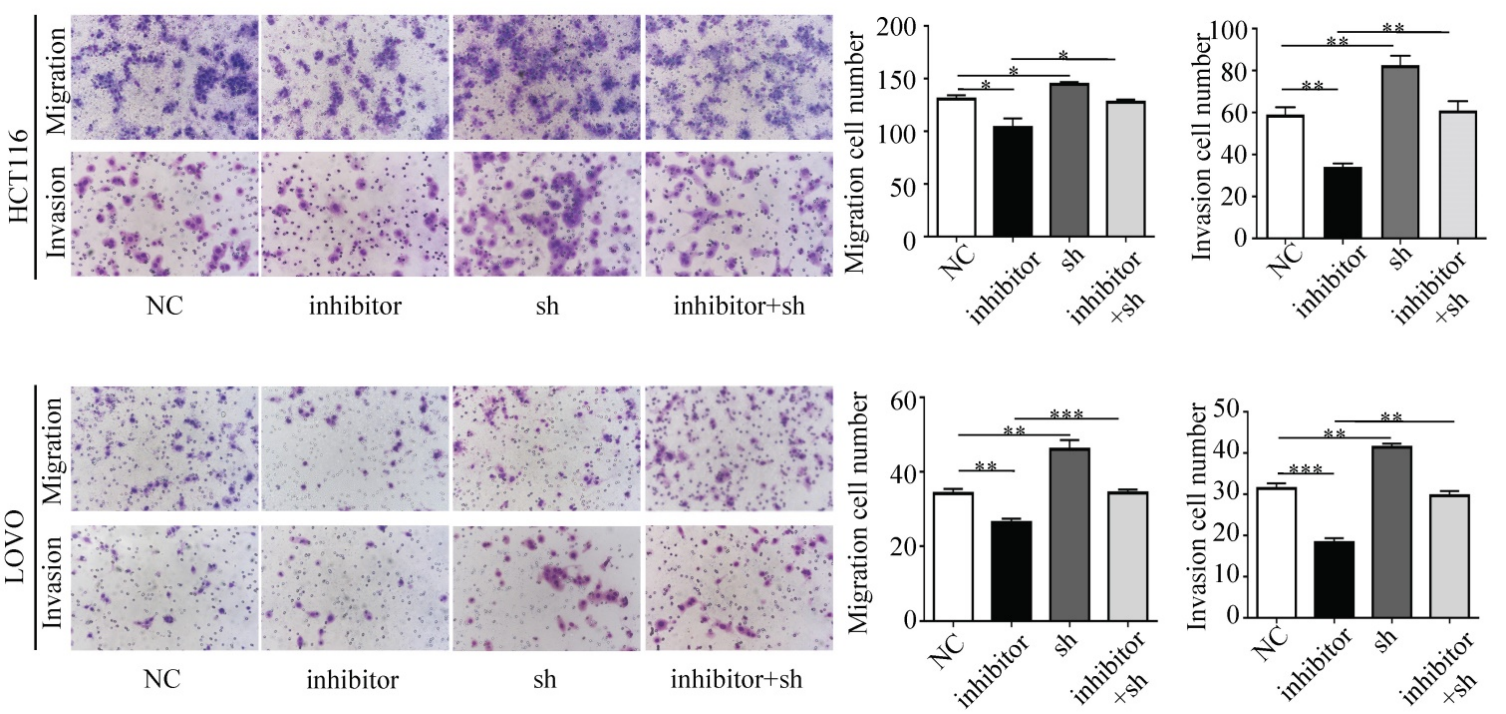
Transwell assay was performed to assess the migration and invasion potency after the expression of miR-17-5p and HSPB2 was down-regulated alone or simultaneously. * P <0.05; ** P <0.01; *** P <0.001.

Next, we performed a rescue experiment in HCT116 and LOVO cells. When miR-17-5p expression was exogenously increased, we verified that overexpressed miR-17-5p could enhance cell viability, colony formation (Figure 7C-D), migration and invasion (Figure 8) as compared with the control group. When miR-17-5p was endogenously silenced, it was reconfirmed that compared to the control group, knockdown of miR-17-5p could reduce cell viability, colony formation (Figure 9C-D), migration, and invasion (Figure 10). We overexpressed HSPB2 in HCT116 and LOVO (Figure 7A-B). CCK8 and colony formation assay revealed that HSPB2 overexpression inhibited CRC cell viability and colony formation compared to the control group (Figure 7C-D). The Transwell assay showed that overexpression of HSPB2 significantly reduced the number of migrating and invading cells compared to the control group (Figure 8). Then HSPB2 expression was knocked down by the shRNA (Figure 9A-B). Compared with the control cells, reduced HSPB2 promoted cell viability and colony formation (Figure 9C-D), and increased the number of migrating and invading cells (Figure 10). In addition, we found that overexpression of miR-17-5p suppressed HSPB2 at both the mRNA and protein levels (Figure 7A-B) and silencing of endogenous miR-17-5p increased HSPB2 at both the mRNA and protein levels (Figure 9A-B). When miR-17-5p and HSPB2 expression were increased simultaneously, the reintroducing HSPB2 caused by co-overexpression attenuated the tumor-promoting effect of overexpressed miR-17-5p, as shown by reduced proliferation (Figure 7C-D), migration and invasion (Figure 8). Simultaneous knockdown of miR-17-5p and HSPB2 revealed that reduced HSPB2 could partially reverse the suppression of proliferation (Figure 9C-D), migration and invasion (Figure 10) caused by miR-17-5p inhibitor.

## Discussion

In this study, we detected the expression of miR-17-5p in 47 pairs of CRC tissue and adjacent normal tissue samples, and the results showed that miR-17-5p was significantly upregulated in CRC tissues and was associated with lymph node metastasis. miR-17-5p is a critical oncogenic miRNA, and the increased expression of miR-17-5p was found to be involved in the development of multiple tumors, including hepatocellular carcinoma^16^, gastric cancer^32^, prostate cancer^33^, ovarian cancer^34^, and breast cancer^35^. miR-17-5p overexpression was also found to inhibit cell proliferation, invasion, and tumor metastasis in certain cancer cell lines^36^. However, it is still necessary to clarify how miR-17-5p regulates the development of CRC. Our research results show that overexpression of miR-17-5p promoted the proliferation, anti-apoptosis, migration and invasion of CRC cells, whereas knockdown of miR-17-5p inhibited cell proliferation, migration and invasion but had no significant effect on apoptosis. Taken together, these results suggest that miR-17-5p may contribute to the development of CRC by promoting certain malignant biological characteristics such as growth and metastasis. miRNA regulates gene expression by binding to the 3'-UTR region of the target gene for translational inhibition or degradation. We performed transcriptome sequencing to obtain differentially expressed genes affected by expression of miR-17-5p. And we infer that HSPB2 is the target of miR-17-5p with the help of miRNA target prediction software. Subsequently, the luciferase reporter assay was used to validate that miR-17-5p directly binds to the 3'-UTR region of HSPB2. This is the first demonstration of miR-17-5p as a tumor miR targeting HSPB2 in CRC.

HSPs can interact with its substrate to keep the denatured protein in a folded state and transform it into a functional conformation as a molecular chaperone^37^, ^38^. Furthermore, HSPs have been reported to be involved in cancer cell proliferation, survival, and apoptosis^39^-^41^. HSPB2 is a novel and unique member of the HSPs family, is located on the 11q22-q23 chromosome^42^, ^43^. According to reports, HSPB2 expression is reduced in esophageal squamous cell carcinoma^26^ and pancreatic cancer, and can inhibit the progression of pancreatic cancer^27^. HSPB2 also inhibits the apoptotic pathway by being concentratedly expressed in breast cancer and inhibiting the activation of caspase-8 in breast cancer^25^. However, there is no report on the impact of HSPB2 on CRC. Our results show that the expression of HSPB2 in CRC is significantly lower than that of paired samples from adjacent normal tissues. Analysis of the CRC data set of TCGA, COAD and READ data set of GEPIA showed that the expression of HSPB2 in cancer tissues was significantly reduced compared with normal tissues. This indicates the potential role of HSPB2 as a CRC suppressor gene and makes our research clinically relevant.

To study the role of miR-17-5p and HSPB2 in the development of CRC, we performed gain and loss-of-function assays. A negative correlation between miR-17-5p and HSPB2 expression was observed. In addition, HSPB2 showed an inhibitory effect on the proliferation, migration and invasion of CRC cells, which is contrary to the phenotype of miR-17-5p. This is important evidence that supports miR-17-5p targeting HSPB2 to promote the CRC. Mutations in p53 will make it lose some anti-cancer functions, and allow the mutants to acquire a series of properties similar to oncogenes, such as accelerating the progress of cancer, enhancing the chemoresistance of cancer cells, and preventing the occurrence of apoptosis^44^-^46^. Studies have shown that there are p53 binding sites in the promoter region of HSPB2, and HSPB2 can promote p53-mediated metabolism by affecting related metabolic genes including TIGA R and SCO2^47^. In addition, HSPB2 can bind to p53 to repair its transcriptional activity, so that the repaired p53 can bind to the tumor suppressor RPRM, BAI-1 and TSAP6 promoter regions, and initiate these three genes expression to inhibit the progress of pancreatic cancer cells^27^. It can be speculated that the function of miR-17-5p as an oncomiR in CRC maybe exerted by inhibiting its target HSPB2 and then affecting the expression of p53 and its related genes. Moreover, a study has shown that HSPB2 in human esophageal squamous cell carcinoma is methylated in a cancer-specific manner, and the expression of HSPB2 is lost or down-regulated^26^. We suppose that there are many regulatory relationships that affect miR-17-5p-HSPB2 in CRC, and these relationships greatly complicate the miR-17-5p-HSPB2 regulatory network.

Some limitations need to be resolved. First, this study mainly focused on the role of miR-17-5p and its target HSPB2 in CRC, so the expression of p53 in cells and tissues was not detected. Second, this study did not systematically study the effect of HSPB2 on endogenous p53 and the confirmation of the miR-17-5p-HSPB2-p53 regulatory axis. Third, only CRC cells were used in the study, and normal colorectal cells were not set as controls. These tasks will be gradually completed in the next experiment.

In summary, this study unravels a novel mechanism for tumor progression through which miR-17-5p promotes CRC cell proliferation, migration and invasion by targeting HSPB2. This suggests that miR-17-5p can be used as a diagnostic and prognostic biomarker for CRC patients. Future studies on miR-17-5p and HSPB2 are warranted to develop novel molecular therapeutic avenues.

## Figures and Tables

**Table 1:** The relationship between the relative expression level of miR-17-5p and clinicopathological variables in 47 patients with CRC.

Clinicopathological data	n	miR-17-5p relative expression	U	P
Gender				
Male	31	6.7371 (3.1734, 10.0152)	233	0.558
Female	16	7.0274 (3.9014, 14.7741)		
Age (years)				
≥68	24	6.7974 (3.2270, 10.4221)	239	0.827
<68	23	7.0274 (3.2852, 9.7473)		
Position				
Colon	22	5.3080 (2.8689, 7.7239)	327.5	0.218
Rectum	25	8.8520 (3.9014, 14.2116)		
Differentiatad degree				
Poorly differentiated	8	9.1324 (8.1624, 10.2390)	86	0.230
Moderately and Highly differentiated	39	6.1988 (3.1362, 10.4831)		
Invasive status				
Penetrating serosa	29	7.1514 (3.5859, 9.7126)	213.5	0.929
Non-penetrating serosa	18	6.1988 (3.0022, 12.6758)		
Lymph node metastasis				
Absence	29	5.5983 (2.9601, 8.8104)	128.5	0.018^*^
Presence	18	8.9922 (5.7549, 14.5365)		
